# Confidence interval for quantiles and percentiles

**DOI:** 10.11613/BM.2019.010101

**Published:** 2018-12-15

**Authors:** Cristiano Ialongo

**Affiliations:** Department of Human Physiology and Pharmacology, University of Rome Sapienza, Rome, Italy

**Keywords:** biostatistics, statistical methods, confidence intervals, extra-analytical phase

## Abstract

Quantiles and percentiles represent useful statistical tools for describing the distribution of results and deriving reference intervals and performance specification in laboratory medicine. They are commonly intended as the sample estimate of a population parameter and therefore they need to be presented with a confidence interval (CI). In this work we discuss three methods to estimate CI on quantiles and percentiles using parametric, nonparametric and resampling (bootstrap) approaches. The result of our numerical simulations is that parametric methods are always more accurate regardless of sample size when the procedure is appropriate for the distribution of results for both extreme (2.5^th^ and 97.5^th^) and central (25^th^, 50^th^ and 75^th^) percentiles and corresponding quantiles. We also show that both nonparametric and bootstrap methods suit well the CI of central percentiles that are used to derive performance specifications through quality indicators of laboratory processes whose underlying distribution is unknown.

## Introduction

Percentiles and quantiles are statistics used for summarizing the relative location of data within a set according to their magnitude and independently from a particular underlying probability distribution (*1*). Owing to this, they are broadly applied in biomedical field where non-normality due to outlier contamination or natural skewness is commonly observed.

Percentiles are also useful tools in the field of quality management to show the distribution of observed performance data and for attributing quality grading and goals in extra-analytical processes through indicators (*2*). A set of central percentiles that partition the population into equally sized ranges of values (*e.g.* the 25^th^, 50^th^ and 75^th^ percentiles collectively known as “quartiles”) are commonly employed to attribute a progressively higher level of performance (*3*). Another quality application concerns establishing reference intervals for interpreting laboratory tests results (*4*). In this scenario, a pair of extreme percentiles, often 0.5^th^ - 99.95^th^ or 2.5^th^ - 97.5^th^ that cut-off 1% or 5% population, respectively, are used to find those subjects whose testing seems to exceed the expected homeostatic variability in a biological parameter (*5*).

In both of these applications, a sample is drawn once from the population to find out the estimate of the true parameter, afterwards the value is repeatedly applied to a number of new individuals or items to identify them. According to theory, any point estimate is bound to its sample by an instant bias that depends on the randomness that occurred at the time of the sampling process (*6*). Therefore, any new observation randomly withdrawn from the same population is not necessarily compatible with the former point estimate because bias was not necessarily the same. Of course, the issue reduces to show that the newly observed value did not differ significantly from the sample estimate and in turn the true population parameter. To this end, it is suitable using the frequentist confidence interval (CI) whereby it is found the range of putative population true value that did not differ from the sample estimate with a confidence level as large as 1-α (*7*, *8*).

To date, the discussion about using CI on extreme percentiles in building reference intervals has spurred investigations and recommendations that have been included in guidelines issued by official bodies and reviewed in books (*9*-*17*). However, little has been done concerning central percentiles, which are pivotal for the undergoing development of the extra-analytical quality field. Therefore, the present work was meant to give a theoretical introduction to the topic, also providing a comparison of the methods suitable for placing CI on percentiles via parametric, non-parametric and resampling approaches. To this end, we made use of numeric simulations for reproducing various conditions commonly encountered in laboratory medicine quality management where the departure from normality is more or less pronounced but the true population distribution is seldom known.

## A technical premise on percentiles and quantiles

First of all, let’s start recalling that the quantile (x_r_) is the r^th^ element within an ordered set of size N whose value is larger than or equal to that of q = r/N elements (*i.e*. x_1_ ≤ x_2_ ≤…≤ x_r_…≤ x_n-1_ ≤ x_n_). According to the frequentist paradigm, the probability (P) that any observation x_i_ within the set has to occur can be defined with respect to x_r_ with the following equation (Eq.):

P(xi ≤ xr) = q (Eq. 1).

If certainty is expressed as 100% of occurrence of observations, then it can be written that p = (100*q)% is the percentile of the dataset (*1*). Since the distinction between percentiles and quantiles reduces to the indexing, then methods discussed in the next sections are equally valid for both of them, even though they are presented using the quantile and thus x_r_.

## The parametric CI method (in the Gaussian case)

Several parametric approaches can be used in order to estimate CI about the sample quantile (P-CI) when the underlying distribution is of the Gaussian kind (*18*). To this concern, if the dataset had average m and standard deviation s, the value x_r_ could be sought straightforwardly via the standardization procedure:

z = (xr - m) / s (Eq. 2).

In fact, rearranging Eq. 2 yields:

xr = m + (z*s) (Eq. 3).

Notably, since n is a sample from the population N, m and s were estimates of the true parameters µ and σ respectively. Accordingly, x_r_ was the estimate (^x_r_) of the population true quantile X_r_ giving the partition R = q/N. Therefore, we can write:

^xr = m + (z*s) (Eq. 4)

Xr = µ + (z*σ) (Eq. 5).

Particularly, Eq. 5 shows that wherever µ and σ were known also the true quantile X_r_ was so.

At this point it is possible to reason concerning the CI on ^x_r_ (*19*). Of course, the estimate ^x_r_ depended on both the sampling error (s) and the true value of the quantile X_r_. If the latter was postulated basing on the assumption of normality and thus was given through the z of the standard normal curve N(0,1) according to Eq. 5 for the given percentile q, then we could summarize the “accuracy” of our finding through the quantity:

V = (^xr - Xr) / s (Eq. 6).

There is a striking similarity between Eq. 6 and the Student t-statistic, and indeed the V-statistic shows how ^x_r_ varies around X_r_ that in turn is how the estimate difference (^x_r_ - X_r_) is distributing. To this regard, the V-statistic was shown to follow a Student t-distribution with n-1 degrees of freedom but with non-null centrality parameter (*19*). This particular condition (termed “non-centrality”) is explained by the evidence that the V-statistic was taken under the alternative hypothesis of non-null difference of the estimate from the true parameter since ^x_r_ was assumed to be biased (*i.e.* deviating) by definition according to Eq. 4 (in contrast, the t-statistic is usually evaluated under the hypothesis of null difference and thus the non-centrality parameter equates 0) (*18*). Accordingly, the distribution of the V-statistic can be used to find out the range that ^x_r_ was expected to lay in with probability of untrue finding equal to α:

P(a ≤ V ≤ b) = 1 – α (Eq. 7).

Therefore, rewriting Eq. 6 in the appropriate manner and substituting for it in Eq. 7, given the non-centrality parameter of the t distribution λ = - z*n^0.5^ (that we will refer to as t_[n-1,λ]_) it can be shown after simplifications:

lower P-CI = [m – (t1-α/2,[n-1,λ]*s*n -0.5)] (Eq. 8)

upper P-CI = [m – (tα/2,[n-1,λ]*s*n -0.5)] (Eq. 9).

Notably, whenever the underlying distribution is known, although not Gaussian (*e.g.* Weibull or lognormal), an appropriate parametric procedure to form CI can be set up likewise (*20*, *21*).

## The non-parametric CI method

Let us begin recalling that for q = r/n, then r = n*q (*e.g.* if q = 0.2 and n = 10 then r = 2) that is simply the number of elements within the subset identified by x_r_.

Supposing that n was withdrawn from a population N, obviously the sample quantity x_r_ depended upon how many observations smaller than or equal to the population X_r_ were actually withdrawn. To this concern, it must me noted that each sample observation x_i_ had chance q to be x_i_ ≤ X_r_ (*i.e.* belonging to the partition of N as large as q*N), and thus it was known *a-priori* regardless of the true value X_r_ (*22*, *23*).

According to the reasoning so far, the chance to find a certain estimate of X_r_ (say ^x_r_) can be reduced to the probability that sampling n consecutive and independent x_i_ produced r findings smaller than or equal to X_r_ when this particular event had an individual chance as large as q. Noteworthy, this is nothing but the probability associated with n binomial trials (*i.e.* success in withdrawing x_i_ ≤ X_r_) and it can be easily sought whereby the binomial distribution with parameters n and q that is Bin(n,q) (*23*).

The first remark is that given n trials and the a-priori probability q of success, the expected outcome with the greatest chance to happen is exactly n*q = r. Therefore, r is the average of the sampling process which produced the dataset n and in turn the estimate ^x_r_. Secondly, random outcomes with k ≠ r, and thus with larger or smaller number of observations x_i_ ≤ X_r_ follow the distribution Bin(n,q).

The latter notion is noteworthy since it is useful for calculating the CI to be placed on x_r_. In fact, let us recall that by definition the CI is the interval that covers the putative true value of a population parameter with confidence 1-α, given the observed sample values. Therefore, given sample size of n and given the *a-priori* probability q, the CI can be stated in terms of realizations of binomial trials for k_L_ ≤ r and k_U_ ≥ r as follows:

P(kL ≤ r ≤ kU) ≈ 1-α (Eq. 10).

Hence, if x_L_ and x_U_ are the observations that cut off as many observations as k_L_ and k_U_ respectively, it can be written that:

P(xL ≤ xr ≤ xU) ≈ 1-α (Eq. 11).

Thus recalling Eq. 1 it yields (*23*):

P(xr ≤ xU) - P(xr ≤ xL) = Bin(xU;n,q) – Bin(xL;n,q) ≈ 1-α (Eq. 12).

Where Bin(x_k_;n,q) is the cumulative binomial distribution that enables the estimation of the probability of having up to k success in n trials given the a-priori probability q.

Several remarks are in order. First, Eq. 12 does not provide an exact coverage as it was for Eq. 7 since Bin(n,q) is a discrete distribution with only n+1 outcomes. More precisely, it tells that each value in the pair (x_L_;x_U_) must correspond to a fraction in the cumulative probability so that their difference is as close as possible to 1-α (and sometimes the symbol “≥” is preferred to “≈”). Therefore, (x_L_;x_U_) must be sought through an iterative procedure that attempts several alternative pairs and compares. Second, Eq. 12 only depends on the realization of q probability in n trials and not on the sample statistics. Therefore, this method does not require any assumption regarding the underlying distribution of data, so that it is regarded as distribution-free or non-parametric (NP-CI).

If sample size is adequate (usually n ≥ 20) it is possible to exploit the so-called normal approximation of the binomial distribution to simplify the procedure (*24*). In fact, it can be stated that:

lower NP-CI = (n*q) – zα/2* ((n*q)*(1 - q))0.5 (Eq. 13)

upper NP-CI = (n*q) + zα/2 * ((n*q)*(1 - q))0.5 (Eq. 14).

Where the term (n*q)*(1-q) in both equations is the standard deviation of the approximated normal distribution and z_α/2_ is the standardized value of the normal quantile (Eq. 2) cutting off the values in either tail whose cumulative probability is less than α/2. The equations above return the size of the subset that they respectively bound so that the rounded value corresponds to the position of the bounding value within the ordered set. For instance, if Eq. 13 yielded “6” then the lower NP-CI would correspond to the observation upper-bounding the subset of the least 6 observations. See Appendix for further details on calculation.

## The resampling-based non-parametric (bootstrap) CI method

A third way for estimating CI about the quantile estimate involves data resampling (*25*). The underlying principle is quite obvious: if the random sample was produced by virtue of the “tendency” to follow a certain distribution in the original population, then the random sampling of the sample itself (*i.e.* the re-sampling) would reasonably replicate the very same tendency (*26*, *27*).

Let us accordingly suppose that the “tendency” for a quantile q was represented by r = n*q, so that in absence of any sampling bias x_i_ ≤ x_r_ was actually x_i_ ≤ X_r_ and the population estimate ^x_r_ would be unbiased. In a real-world sample, randomness bias adds to the tendency in the population so that how many observations that were truly x_i_ ≤ X_r_ remains unknown and ^x_r_ is only a “particular” realization of X_r_. Nonetheless, resampling the sample adds further bias to the former sampling process, so that the “second-generation” estimate ^x_r_ would be differently biased.

If what is stated so far holds true for a single resample, actually randomness in resampling means that resampling bias may even be negative with respect to the original sampling bias. Therefore, if the resampling process was repeated a very large number of times (*i.e.* ≥ 1,000), then a distribution of bias about ^x_r_ would arise (*27*). Therefore, the resampling distribution would show different proportions of observations x_i_ ≤ x_r_, and thus of x_i_ ≤ X_r_, thereby allowing to gain information on the putative values for the true population parameter X_r_. Notably, this is somehow similar to what seen previously for the NP-CI, where the binomial distribution was used to show alternative partitioning for the same sampling size n and the *a-priori* probability q.

The way in which it is used the information about the shape of the resampling distribution of X_r_ grounds the so-called non-parametric Bias Corrected-accelerated bootstrap method for building CI (BCa-CI) (*28*). Briefly, if α/2 and 1-α/2 are the percentiles of the resampling distribution providing 1-α coverage, indeed their centring and thus accuracy about the true population parameter is correct only when the resampling distribution is unbiased. Otherwise, it is necessary to adjust their “position” shifting the percentile boundaries from the original points to new ones that are the following:

lower BCa-CI = Φ (^z0 + ((^z0 + zα) / (1 - ^a*(^z0 + zα))) (Eq. 15)

upper BCa-CI = Φ (^z0 + ((^z0 + z1-α) / (1 - ^a*(^z0 + z1-α))) (Eq. 16).

where Φ denotes the cumulative standard normal distribution, z_α_ and z_1-α_ are the quantiles of the standard normal distribution, ^z_0_ and ^a are the parameters accounting respectively for resampling bias (*i.e.* the standardized proportion of ^x_r_ > x_r_) and skewness (*i.e.* change of variance across the distribution from tail to tail when they are not symmetric about ^x_r_) (*25*, *28*). Of course, the expected coverage probability remains 1-α. More comprehensive explanation on bootstrap resampling methods and related procedures to build CI can be found elsewhere (*29*-*32*).

## Simulation study

The basic features concerning CI are represented by: a) the actual coverage probability or interval accuracy, b) the interval width and c) the interval centring or asymmetry (*i.e.* the ratio between the distances of the estimate from each of the boundaries). For the sake of conciseness we concerned mainly with point “a” as it is the one that should primarily be considered when making comparisons through different methods. Nonetheless, points “b” and “c” were considered only when the actual coverage probability was close to the nominal goal of 1-α.

To this end, we proceeded as follows: a) a theoretical model represented by the generalized 3-parameter lognormal distribution was used to generate sets of artificial data each featured by a combination of location (α = 0.5, 1.0, 2.0 and 3.0) scale (β = 0.05, 0.2, 0.5 and 1.2) and threshold (γ = 0) in order to reproduce a particular degree of asymmetry and tailing (*i.e.* skewness) with only positive values (X ≥ 0); b) for each set it was generated 3 batches of 100 samples sized n = 20, n = 60 and n = 120 respectively; c) for each combination of parameters the 2.5^th^, 25^th^, 50^th^, 75^th^ and 97.5^th^ percentiles were computed whereby the corresponding theoretical lognormal function obtaining the “true” or population value and the actual coverage probability was the proportion of sample CI that contained it. Particularly, CI were computed whereby Eq. 8 and Eq. 9 for P-CI, Eq. 13 and Eq. 14 for NP-CI, and Eq. 15 and Eq. 16 for BCa-CI. In order to evaluate and make appropriate comparisons on performance, the advisable optimum was represented by covering at least the 1-α nominal value which was set equal to 95% in this study.

All the calculations were performed using Excel 2010 (Microsoft Corp., Redmond, USA), except for BCa that was performed using SPSS 20.0 (IBM Corp., Armonk, USA) and data generation that was carried out exploiting the pseudo-random number generator embedded in Minitab 17 (Minitab Inc., State College, USA).

An electronic spreadsheet based on Microsoft Excel framework is provided in Supplementary material in order to allow automatic calculations of P-CI and NP-CI for up to 500 sample data, plus an additional file with Worked examples showing practical applications in "real-world" scenarios of the methods here described.

## Results

### Data modelling

**Figure 1:**
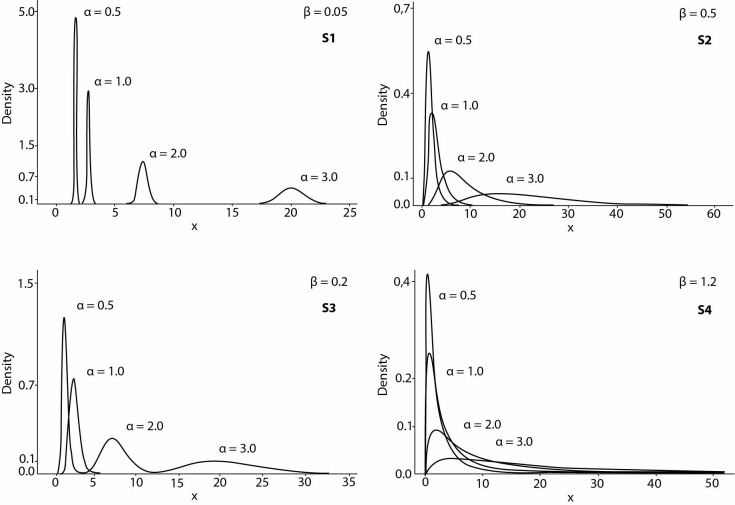
Theoretical frequency distribution of the simulated data generated by the 3-parameter lognormal probability density function. By varying scale (β) and location (α) parameters with threshold (γ) fixed at 0 it is possible to reproduce different combinations of asymmetry, tailing and kurtosis (flatness) that give rise to the testing conditions described in the result section as S1 - S4. In general, increasing α gives more flatness to the shape while β more asymmetry, whereas γ relatively affects the degree of left-fronting since it constrains the distribution of data to a certain lower bound.

The combinations of scale and location parameters gave rise to the following data models (Figure 1): S1) for β = 0.05 the shape was almost Gaussian and thereby equivalent to a normal distribution with coefficient of variation CV ≈ 5% and shape changing from leptokurtic (*i.e.* peaky) by α = 0.5 to platykurtic (*i.e.* flat) by α = 3.0; S2) for β = 0.2 the shape was almost Gaussian and thereby equivalent to a normal distribution with coefficient of variation CV ≈ 20% and same changes in shape seen for S1 at the variation of α; S3) for β = 0.5 the shape was right-skewed and changed from minimal right-tailed and platykurtic by α = 0.5 to heavily right-tailed and platykurtic by α = 3.0; S4) for β = 1.2 the shape was left-fronted (*i.e*. almost no left tail) and changed from leptokurtic with short right-tailing by α = 0.5 to platykurtic long heavy right-tailing by α = 3.0.

### CI accuracy

**Figure 2:**
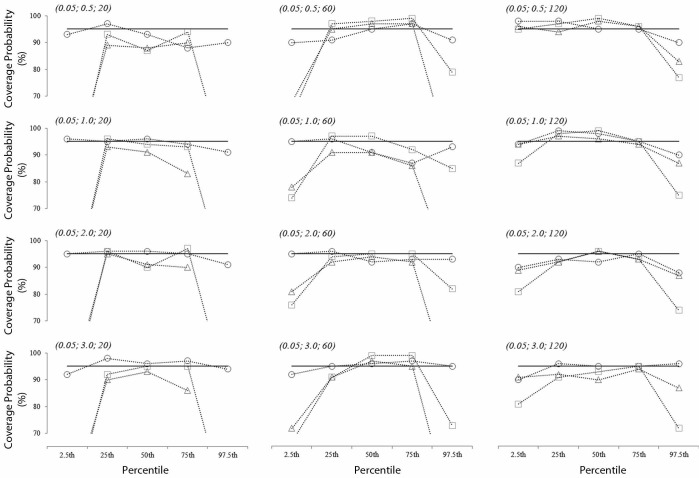
Actual coverage probability in the quasi-Gaussian model of data with low CV% (S1). The coordinates in brackets (β;α;n) represent scale (β) and location (α) parameters of the lognormal distribution (with 0 threshold) generating the artificial data for a given sample size (n). The solid black line shows the desired coverage probability (95% in this case) that is achieved when the symbol representing the parametric-CI (circle), nonparametric-CI (square) or BCa-CI (triangle) lays on it.

Analysis showed four different scenarios with respect to the actual coverage probability. When shape was the kind of S1 (Figure 2), the P-CI performed better than both NP-CI and BCa-CI for n = 20 as well as n = 60, while the three were almost equivalent for n = 120. However, the P-CI was the only one to provide adequate coverage probability for both the extreme (2.5^th^ and 97.5^th^) and the central percentiles (25^th^, 50^th^ and 75^th^).

**Figure 3:**
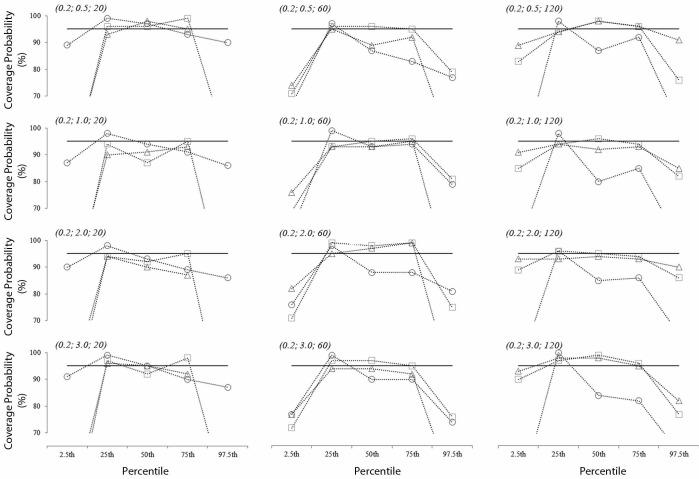
Actual coverage probability in the quasi-Gaussian model of data with high CV% (S2). The coordinates in brackets (β;α;n) represent scale (β) and location (α) parameters of the lognormal distribution (with 0 threshold) generating the artificial data for a given sample size (n). The solid black line shows the desired coverage probability (95% in this case) that is achieved when the symbol representing the parametric-CI (circle), nonparametric-CI (square) or BCa-CI (triangle) lays on it.

When the shape was the kind of S2 (Figure 3), the three CI-building procedures performed almost equally adequate for the central percentiles regardless of n, while only the BCa-CI was close to the goal of 1-α for the extreme percentiles when sample size was large (*i.e.* n = 120).

**Figure 4:**
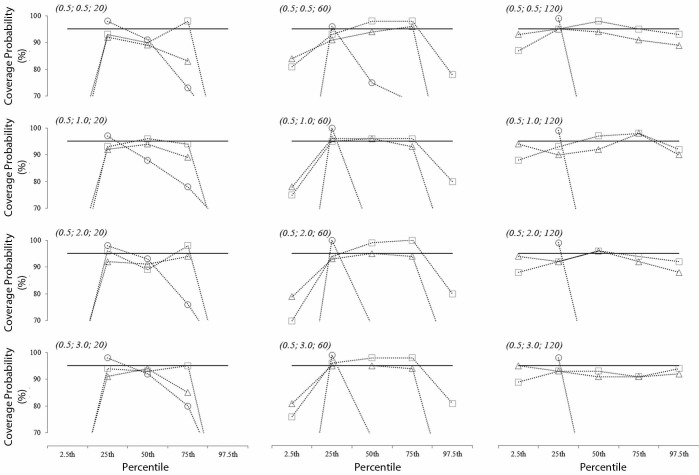
Actual coverage probability in the right-skewed model of data with right tailing (S3). The coordinates in brackets (β;α;n) represent scale (β) and location (α) parameters of the lognormal distribution (with 0 threshold) generating the artificial data for a given sample size (n). The solid black line shows the desired coverage probability (95% in this case) that is achieved when the symbol representing the parametric-CI (circle), nonparametric-CI (square) or BCa-CI (triangle) lays on it.

When the shape was the kind of S3 (Figure 4), the P-CI resulted in unsatisfactory performance regardless of n for any percentile except for the 25^th^. On the contrary, both NP-CI and BCa-CI performed satisfactorily for the central percentiles, but their coverage of the extreme ones was always slightly below the goal of 1-α even for n = 120.

**Figure 5:**
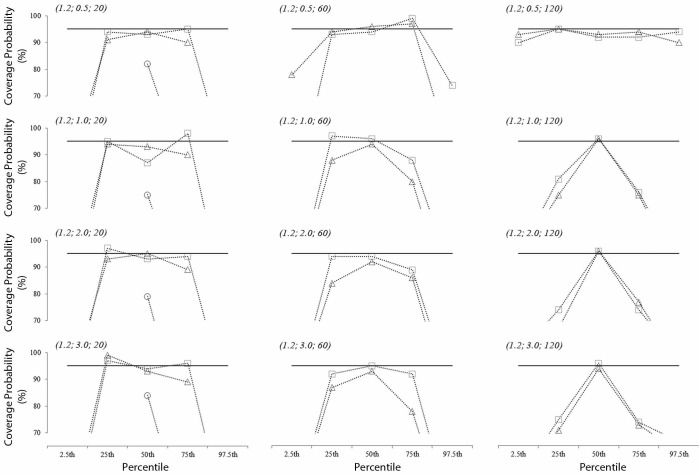
Actual coverage probability in left-fronted model of data with left fronting (S4). The coordinates in brackets (β;α;) represent scale (β) and location (α) parameters of the lognormal distribution (with 0 threshold) generating the artificial data for a given sample size (n). The solid black line shows the desired coverage probability (95% in this case) that is achieved when the symbol representing the parametric-CI (circle), nonparametric-CI (square) or BCa-CI (triangle) lays on it.

Finally, when the shape was the kind of S4 (Figure 5), the P-CI always returned unreliable boundaries (*e.g.* negative values) or poor coverage depending on n. Also in this case, both NP-CI and BCa-CI always provided satisfactory performance for the 50^th^ percentile regardless of the data shape while the capability to cover all three central percentiles was gained just for n = 120 and platykurtic distribution. Notably, extreme percentiles were never adequately covered regardless of shape and sample size.

### CI width and shape

Whit respect to extreme percentiles, width and shape of P-CI was incomparable with NP-CI and BCa-CI because of poor accuracy in the latter two methods.

With respect to central percentiles, for samples generated according to models S1 and S2, all three building methods returned fairly symmetric intervals, which were sensibly narrower for the P-CI. However, when data where generated according to model S3 and S4, data skewness reflected into proportionally increasing width and symmetry by 25^th^ to 75^th^ percentile for both NP-CI and BCa-CI.

**Figure 6:**
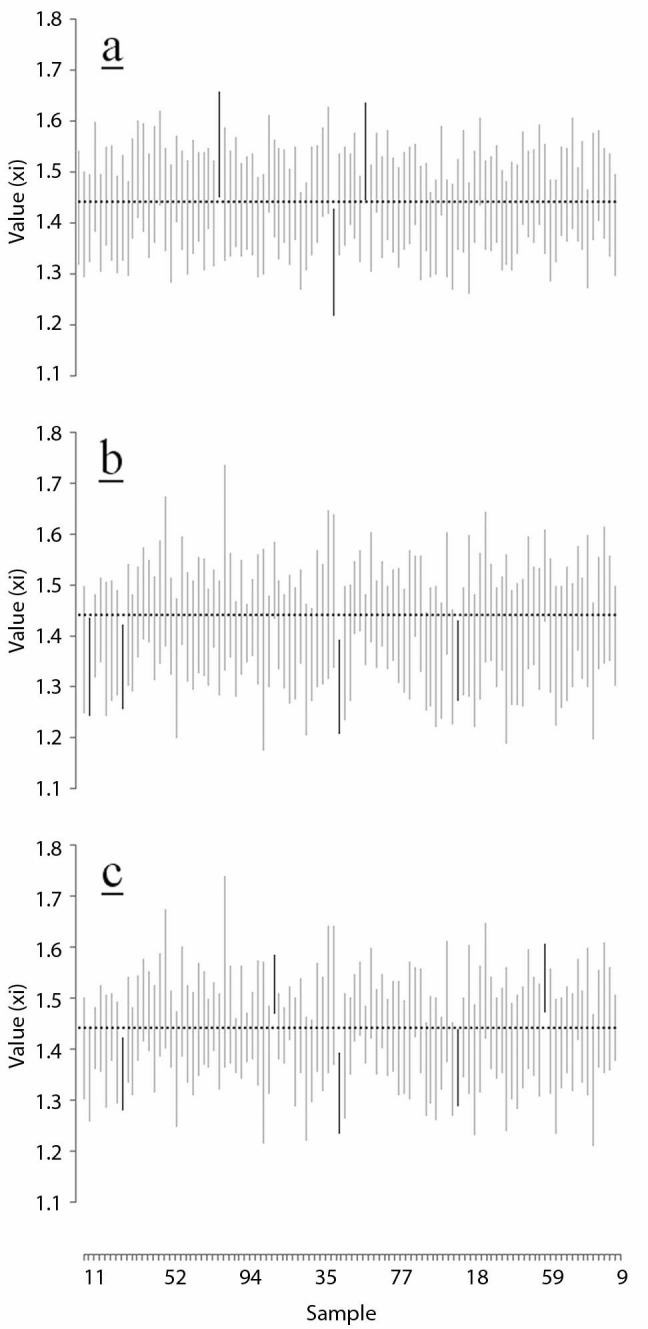
Example of actual coverage probability (CI accuracy) pattern by numeric simulation. It is shown an example of the 25^th^ percentile CI for sample size n = 60 and parameters of the lognormal distribution scale β = 0.2, location α = 0.5, threshold γ = 0, computed according to the parametric (a), nonparametric (b) and BCa (c) method. Dotted grey line is the true population 25^th^ percentile while solid black lines are the CI that do not contain it.

It is remarkable that there was negligible difference in the boundaries returned by NP-CI and BCa-CI for a given percentile, shape and size of the sample up to n = 120. That similitude was preserved up to the size n = 320 (data not shown), even though BCa-CI resulted slightly narrower despite being equally centred to NP-CI (Figure 6).

## Discussion

In this work, we described three methods for building CI on quantiles and percentiles and investigated their performance regarding different data shape and sample size. Foremost, we observed that NP-CI and BCa-CI almost equally behaved in all the situations herein explored, while P-CI was evidently something different because explicitly bounded to the normality of data. However, the major difference was due to the way the three procedures produced the CI boundaries. Indeed, the P-CI relied upon the distribution of the sample statistic and thus an external set of values derived by a theoretical distribution (the non-central t). By contrast, NP-CI as well as BCa-CI used observed values, and precisely the NP-CI did it directly by picking up value from the sample according to an alternative partitioning while the BCa-CI did it indirectly by recreating the alternative partitioning through resampling and picking up the values afterwards. Thereby, NP-CI and BCa-CI were constrained to return boundaries that were always within the range of the sample values.

Such technical aspects explain our major findings. Indeed, NP-CI and BCa-CI never returned CI boundaries with negative sign, which is crucial since we supposed to deal only with positive quantities. However, the analysis of the extreme percentiles demonstrated that their performance was dramatically affected by the sample size for they required n = 120 or even larger depending on the degree of skewness and tailing to achieve acceptable accuracy (*i.e*. ≈ 90%) (*33*, *34*). In contrast, P-CI were accurate yet by n = 20 where the shape was close to normality (*e.g.* S1), showing the better accuracy of parametric approach (*18*, *22*, *33*). Notably, these findings seem to be contrasting with the recommendation of the International federation of clinical chemistry (IFCC) according to which CI for RI should be computed by means of bootstrapping (*14*). In our opinion, this can be explained considering that IFCC aimed at preventing inappropriate use of parametric techniques by favouring robustness of the procedure over accuracy of the method. In fact, in order to get reliable P-CI it is necessary to satisfy three conditions: 1) that the underlying distribution of data is correctly ascertained, 2) that the fitly P-CI procedure is available, and 3) that the parametric method is appropriately chosen for that distribution. The reader can refer to the worked examples provided in Supplementary materials for a more practical insight also preliminary data analysis. In this regard, we feel to suggest the Anderson-Darling as it is a goodness-of-fit test that quantifies the degree of deviation from normality whereby the AD statistics. Nonetheless, we also recommend using a normality plot to visually inspect potential local deviations in the body or tail of the data distribution, and thus Percentile-Percentile plot or Quantile-Quantile plot for central or extreme percentiles respectively.

NP-CI and BCa-CI were always fairly accurate with respect to the central percentiles, and in quasi-normal samples (*e.g.* S1) they had comparable performance to P-CI although producing slightly wider intervals and thus being less conservative (*22*). For n ≤ 120, negligible difference was found in the boundaries returned by NP-CI and BCa-CI respectively. Of course, in other models of skewness (*e.g*. logistic, log-logistic or Weibull), the location of data especially in small sized samples may produce some more pronounced deviations due to the different weight that tailing gains in the distribution shape. However, we are confident that the non-parametric method is actually suitable in the common practice since it is readily computable by means of electronic spreadsheets (as for instance the one provided in Supplementary Material).

In conclusion, CI should be included for percentiles whenever they are intended as estimates of a population parameter or a criterion to be applied outside the sample where they were estimated. To this end the theoretical distribution underlying the data structure should be always investigated in order to facilitate the choice of the more accurate and conservative parametric CI. In this regard, a statistical test able to quantify the degree of deviation together with a normality plot should be always used in order to assess the compliance of data with the available methods. In the case that parametric methods were not applicable or available, NP-CI and BCa-CI should be equally trusted for the central percentiles, whereas for the extreme percentiles the choice should be based on careful evaluation of the degree of skewness and the density of data in the tails of the distribution.

## Supplementary material

Work Examples

## Appendix

### Finding out percentiles of the non-central t distribution

The most challenging part of computing the P-CI is finding the value of the non-central t distribution. Actually, this is unavailable through Microsoft Excel (unless third-part add-ins are used) as well as some statistical packages most familiar in the biomedical field (*e.g.* Medcalc). Fortunately, the “Keisan” (Casio Computers Co., Tokyo, Japan) offers high-precision on-line calculation from 6 up to 50 significant figures (https://keisan.casio.com/exec/system/1180573219). In Keisan the computation allows only λ ≥ 0 whereas the P-CI can require a negative value. To this concern, recalling that λ is a factor shifting and skewing the central t distribution away from its symmetry about 0, it is easy to understand how non-central t percentiles can be simply flipped and sign-inverted to satisfy symmetry in the negative λ. Hence, placing a = α/2 and b = 1 - α/2 are the percentiles for λ, then for - λ they turn into a’ = - (1 - a) and b’ = - (1 - b) and thus a’ = - (1 - α/2) and b’ = - (α/2). For instance, given λ = 3 and ν = 20 it yields a = 1.027 and b = 5.663, then for λ = - 3 it turns into a’ = - 5.663 and b’ = - 1.027.

### Practical issues in calculating NP-CI for extreme quantiles

Because Bin(n;q) is finite, calculation for extreme percentiles boundaries may lead to unreal results in small samples. For instance, with N = 20 the index of the lower boundary for the 2.5^th^ percentile and the 95% coverage probability is actually - 1 if data are approximately normal. Thus, in order to form this CI in the appropriate manner, one may choose to set arbitrarily the lower boundary to the least observed value in the sample. Of course this way it is cut off part of the actual coverage probability that is bound to be systematically smaller than the theoretical value unless the sample size is adequately large (N > 300 by calculation). To this end there are other methods for computing NP-CI (although not via simple electronic spreadsheet) that overcome this limitation especially for small sized samples (*22*, *33*, *35*).
