# Supplementary material for: Confidence interval for quantiles and percentiles
Source: Biochem Med (Zagreb). 2018 Dec 15;29(1):010101. doi: 10.11613/BM.2019.010101 (PMC6294150; doi:10.11613/BM.2019.010101)
Supplement: Supplementary file 1 — Work Examples [file bm-29-1-010101-S1.pdf]

EXAMPLE 1 – extreme percentiles of skewed data with custom formulae

In a study concerning the assessment of local reference interval in a clinical laboratory, 262 healthy individuals were selected and sampled to test the serum aspartate-aminotransferase (AST)*. Data are summarized in the graph below:

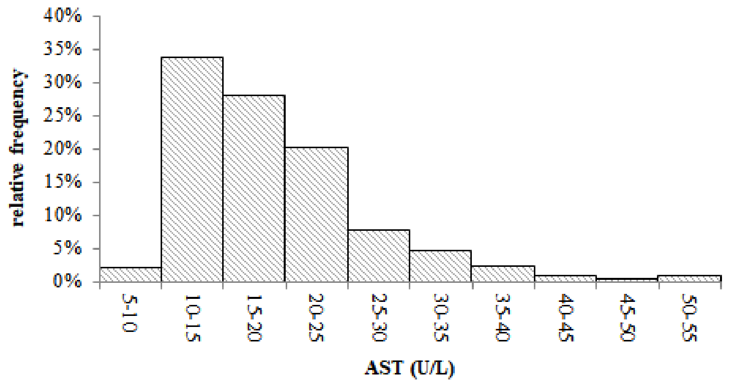

As the skewness was clearly observed, the data manager opted to apply distribution-free analysis in order to find out 2.5th and 97.5th percentiles in the sample and their respective CI. To this end he carried out BCa analysis of 1,000 resamples with the following results:

BCa-CI (U/L)		
**percentile**	**estimate**	**95% CI**
2.5th	10.1	9.6 – 10.5
97.5th	37.1	34.9 – 44.4

Alternatively, the data manager may use the NP-CI method typing in by himself the formulae in a custom electronic spreadsheet without any embedded statistical functions as follows:

1) Data are ordered and progressively numbered, as given below

**Indexed (non-ranked) values of collected AST (U/L)**													
index		index		index		index		index		index		index	
1	8.8	*39*	12.6	*77*	14.2	*115*	16.3	*153*	18.9	*191*	21.9	*229*	28.3
2	9.3	*40*	12.6	*78*	14.2	*116*	16.3	*154*	18.9	*192*	22.1	*230*	28.3
3	9.4	*41*	12.7	*79*	14.3	*117*	16.4	*155*	19.0	*193*	22.2	*231*	28.5
4	9.8	*42*	12.7	*80*	14.4	*118*	16.4	*156*	19.0	*194*	22.3	*232*	28.7
5	9.9	*43*	12.7	*81*	14.4	*119*	16.4	*157*	19.1	*195*	22.4	*233*	28.8
6	10.0	*44*	12.8	*82*	14.4	*120*	16.5	*158*	19.1	*196*	22.5	*234*	28.9
7	10.1	*45*	12.8	*83*	14.5	*121*	16.5	*159*	19.4	*197*	22.6	*235*	28.9
8	10.1	*46*	12.9	*84*	14.5	*122*	16.7	*160*	19.4	*198*	22.8	*236*	29.1
9	10.4	*47*	13.0	*85*	14.6	*123*	17.0	*161*	19.5	*199*	22.8	*237*	29.2
10	10.4	*48*	13.1	*86*	14.7	*124*	17.0	*162*	19.8	*200*	22.8	*238*	29.6
11	10.4	*49*	13.1	*87*	14.8	*125*	17.0	*163*	19.8	*201*	23.0	*239*	29.9
12	10.5	*50*	13.3	*88*	14.8	*126*	17.0	*164*	19.9	*202*	23.0	*240*	30.1
13	10.5	*51*	13.3	*89*	14.8	*127*	17.1	*165*	19.9	*203*	23.1	*241*	30.1
14	10.7	*52*	13.3	*90*	14.8	*128*	17.1	*166*	19.9	*204*	23.3	*242*	30.5
15	10.8	*53*	13.3	*91*	14.9	*129*	17.1	*167*	20.2	*205*	23.4	*243*	30.6
16	10.9	*54*	13.4	*92*	14.9	*130*	17.2	*168*	20.2	*206*	23.5	*244*	30.8
17	11.0	*55*	13.4	*93*	14.9	*131*	17.4	*169*	20.3	*207*	23.5	*245*	30.8
18	11.0	*56*	13.5	*94*	15.0	*132*	17.4	*170*	20.3	*208*	23.6	*246*	31.7
19	11.1	*57*	13.5	*95*	15.1	*133*	17.4	*171*	20.4	*209*	23.7	*247*	31.8
20	11.2	*58*	13.5	*96*	15.2	*134*	17.5	*172*	20.4	*210*	23.8	*248*	33.1
21	11.2	*59*	13.6	*97*	15.3	*135*	17.5	*173*	20.5	*211*	23.8	*249*	34.2
22	11.2	*60*	13.7	*98*	15.3	*136*	17.6	*174*	20.5	*212*	23.8	*250*	34.4
23	11.3	*61*	13.7	*99*	15.4	*137*	17.7	*175*	20.5	*213*	24.0	*251*	34.5
24	11.3	*62*	13.7	*100*	15.5	*138*	17.9	*176*	20.7	*214*	24.2	*252*	35.4
25	11.3	*63*	13.8	*101*	15.5	*139*	18.0	*177*	20.7	*215*	24.3	*253*	35.5
26	11.4	*64*	13.8	*102*	15.5	*140*	18.2	*178*	20.7	*216*	24.4	*254*	36.0
27	11.4	*65*	13.8	*103*	15.6	*141*	18.3	*179*	21.0	*217*	24.6	*255*	36.2
28	11.4	*66*	13.8	*104*	15.6	*142*	18.3	*180*	21.1	*218*	24.7	*256*	38.0
29	11.5	*67*	13.8	*105*	15.6	*143*	18.4	*181*	21.3	*219*	24.9	*257*	38.7
30	11.8	*68*	13.9	*106*	15.6	*144*	18.6	*182*	21.3	*220*	25.4	*258*	40.2
31	11.9	*69*	13.9	*107*	15.7	*145*	18.6	*183*	21.3	*221*	26.2	*259*	43.7
32	11.9	*70*	13.9	*108*	15.7	*146*	18.6	*184*	21.4	*222*	26.4	*260*	45.4
33	12.1	*71*	14.0	*109*	15.8	*147*	18.7	*185*	21.4	*223*	26.5	*261*	50.3
34	12.1	*72*	14.1	*110*	15.8	*148*	18.7	*186*	21.5	*224*	26.6	*262*	54.4
35	12.2	*73*	14.1	*111*	15.8	*149*	18.7	*187*	21.5	*225*	27.2	/	/
36	12.3	*74*	14.1	*112*	16.1	*150*	18.8	*188*	21.6	*226*	27.3	/	/
37	12.5	*75*	14.2	*113*	16.2	*151*	18.8	*189*	21.7	*227*	27.5	/	/
38	12.5	*76*	14.2	*114*	16.2	*152*	18.9	*190*	21.8	*228*	27.6	/	/

2) Recalling that the quantile corresponding to the 2.5th percentile is q=0.025 according to Eq. 1, and that the z-score used for the 95% CI is 1.96, applying Eq. 13 and Eq. 14 it yields:

   a. Lower NP-CI = $(262*0.025) – 1.96*((262*0.025)*(1 - 0.025))^{0.5} = 2$

   b. Upper NP-CI = $(262*0.025) + 1.96*((262*0.025)*(1 - 0.025))^{0.5} = 12$

3) Recalling that the quantile corresponding to the 97.5th percentile is q=0.975 according to Eq. 1, applying Eq. 13 and Eq. 14 it yields:

   a. Lower NP-CI = (262*0.975) – 1.96* ((262*0.975)*(1 - 0.975))$^{0.5}$ = 250

   b. Upper NP-CI = (262*0.975) + 1.96*((262*0.975)*(1 - 0.975))$^{0.5}$ = 260

The figure below is the screen capture of the actual electronic spreadsheet used for the computations described in the previous lines.

P9 *fx*

	A	B	C	D	E	F	G
1	*sample size*	262					
2	*percentile*	0.025	0.975				
3	*z*	1.96					
4							
5	*lower CI index - 2.5th ptile*	**2**	=ROUND((B1*B2)-B3*((B1*B2)*(1-B2))^0.5;0)				
6	*upper CI index - 2.5th ptile*	**12**	=ROUND((B1*B2)+B3*((B1*B2)*(1-B2))^0.5;0)				
7							
8	*lower CI index - 97.5th ptile*	**250**	=ROUND((B1*C2)-B3*((B1*C2)*(1-C2))^0.5;0)				
9	*upper CI index - 97.5th ptile*	**260**	=ROUND((B1*C2)+B3*((B1*C2)*(1-C2))^0.5;0)				
10							
11							

4) Hence in the second table it can be picked up the 2nd and 12th indexed values as well as the 250th and 260th indexed values to form the 95% CI of the 2.5th and 97.5th percentile respectively:

The results for the NP-CI method are summarized in the following table:

NP-CI (U/L)		
**percentile**	**estimate**	**95% CI**
2.5th	10.1	9.3 – 10.5
97.5th	37.1	34.4 – 45.4

The comparison between the CI provided by the two methods is shown in the graphs below for the 2.5th and the 97.5th percentile respectively:

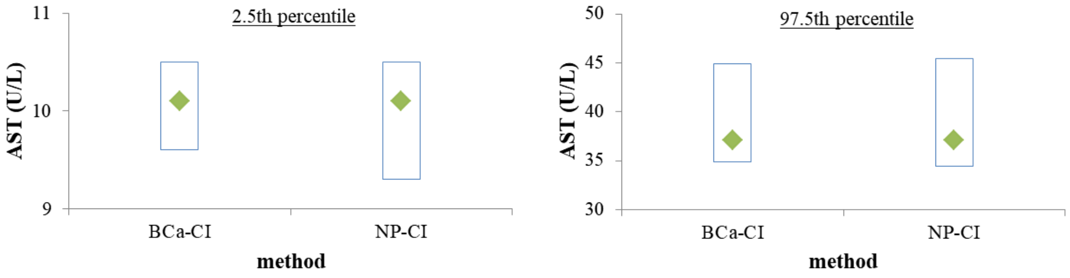

As it can be seen, the difference between the results from the two methods is trivial.

**NOTE: the generation of synthetic AST data was carried out in order to approximate the results shown by Ceriotti et al., Clin Chem Lab Med 2010;48(11):1593-1601, Table 5. To this end, the lognormal modelling was adopted and in particular, the actual generating function had the following parameters: location=2.5, scale=0.5, threshold=5 (threshold was set equal to the least concentration that can be measured with a common chemistry analyser). The corresponding theoretical distribution with 2.5th and 97.5th percentile is shown below. As it can be seen in the second and third table provided before, both BCa-CI and NP-CI covered the true population percentiles.*

It must be remarked that the actual sample could not be normalized whereby a log transformation although generated by a lognormal distribution, as shown by the Box-Cox variance stability plot. In fact, it shows that the power scale (λ) to be applied to data is -0.5, and thus it is a reciprocal square root transformation. Therefore, whenever the data manager had considered log-normalize data basing on historical and literature knowledge about AST distribution, he would actually have produced misleading results as shown by the graph, which represents the Quantile-Quantile plot of log-normalized sample and the Anderson-Darling test against normality. The blue dots outside the red ribbon (representing 95% CI of agreement) bending at the extremes of the plot show a significant loss of normality in the tails of the data distribution in correspondence to the 2.5th *and the 97.5*th *percentiles. In fact, the Anderson-Darling test shows P-Value <0.05 that is significant of non-normality in this case.*

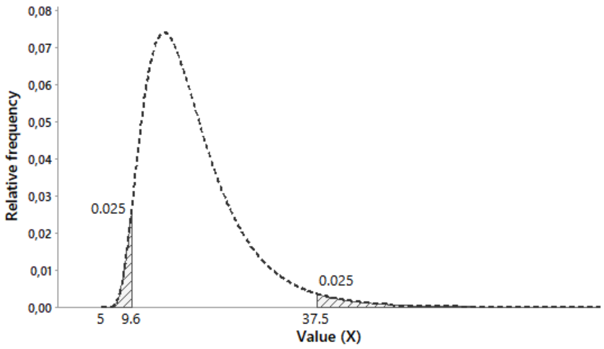

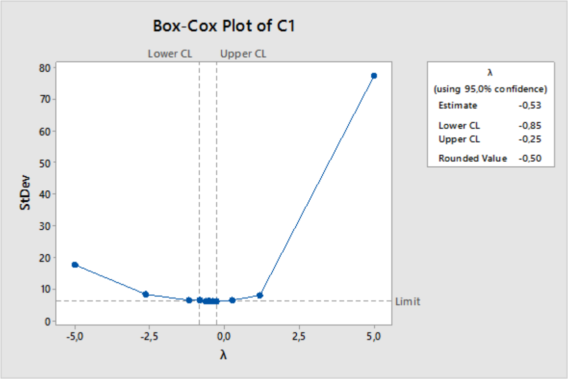

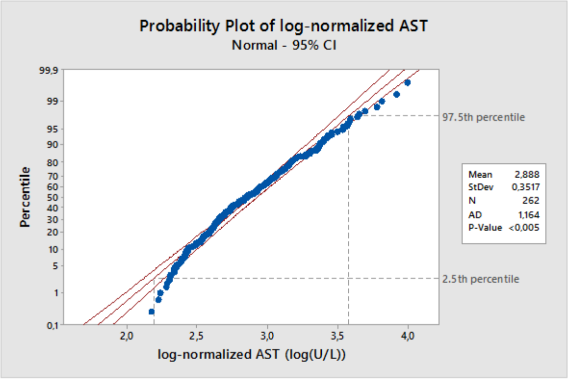

EXAMPLE 2 – central percentiles of quasi-Gaussian data with automated spreadsheet

In an External Quality Assurance (EQA) programme exercise it was collected data of the monthly median turnaround time (TAT) in minutes for red blood cell count (RBCC) from 48 participating laboratories:

**Median TAT of participants**							
14.7	10.7	14.0	18.4	19.8	13.9	9.7	19.1
16.9	15.6	14.8	15.7	8.8	18.1	9.5	12.7
15.9	9.8	13.7	12.5	13.5	10.2	11.2	12.4
19.2	6.1	12.9	17.7	19.8	17.2	10.0	17.0
13.9	12.9	10.8	23.1	13.3	12.5	9.0	16.0
16.4	15.9	13.0	10.0	18.5	22.2	16.0	12.6

The sample 25th, 50th and 75th percentiles in minutes were computed and used to grade the level of timeliness among participants as follows:

**Data analysis**		
percentile	estimate	grade
25th	12.1 min	*improving*
50th	13.9 min	*adequate*
75th	16.9 min	*optimal*

The data manager wishes to display the 95%CI on the sample percentiles to enhance the accuracy of EQA exercise allowing the participants to locate themselves within the quality ladder. To this end, he decides to apply available methods and first he proceeds to assess whether the sample of median TAT was normally distributed by means of the Anderson-Darling test. The results are the following:

**Test of normality**	
average	14.4 min

standard deviation	3.7 min
N	48
AD statistics	0.252
P-Value	0.724

Because the data manager is interested in central percentiles he decides to use the Percentile-Percentile plot to visually inspect local deviations from normality that may affect the centre of the distribution of the data:

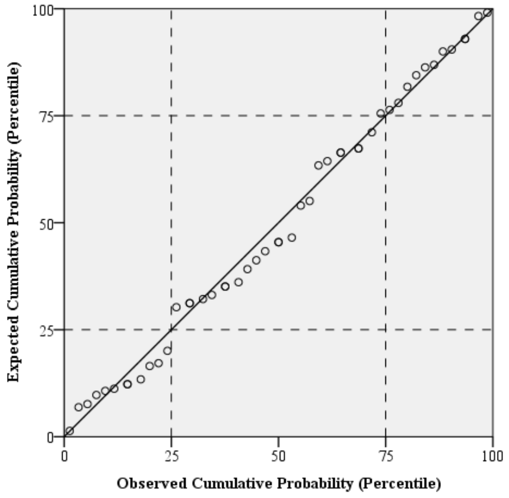

Considering the acceptable agreement with the normal distribution, the data manager applies the method for the P-CI using the "**P-CI_NP-CI_CALCULATOR.xlsx**" (see Supplementary file) as follows:

1) copy and paste into the "data" column the sample data that were already ordered increasingly
2) fill in the "MANUAL INPUT" fields of "unit of measure" (❶), "sought percentile" (❷) and "level of confidence" (❸) with the required specifications, *e.g.* "minutes", "75" and "95" respectively

NOTE: the spreadsheet automatically returns the Z-score of the sought percentile using the NORMSINV function of Microsoft Excel that applies the probit function Φ^{-}

^{1}(p); the result is displayed in the "AUTOMATIC" field under the "PARAMETRIC (GAUSSIAN) CI" panel

3) find out the non-centrality parameter λ of the non-central t distribution in the corresponding "*OUTPUT FOR KEISAN*" field *"①"* under the "PARAMETRIC (GAUSSIAN) CI" panel whose result is λ = 4.673

NOTE: the electronic spreadsheet displays a λ value that is always positive and thus not the actual one since the Keisan calculator allows only λ ≥ 0. Therefore, the user must only copy the "OUTPUT FOR KEISAN" filed "①" and past as it is in the corresponding field of the web application

4) calculate whereby the web application Keisan described in Appendix A the 2.5^{th} and 97.5^{th} percentiles of the non-central t distribution copying the values found in the "OUTPUT FOR KEISAN" fields numbered "①" and "②" plus the typing in the values "0.025" or "0.975" in order to obtain the following results:
   a. Non-central t 2.5^{th} percentile (*i.e.* $t_{\alpha/2,[n-1,\lambda]}$) = 3.996
   b. Non central t 97.5^{th} percentile (*i.e.* $t_{1-\alpha/2,[n-1,\lambda]}$) = 5.478
5) type in directly from the Keisan application the results above into the "MANUAL INPUT" fields numbered "❹" and "❺" in order to complete the calculations for the 95% P-CI according to Eq. 8 and Eq. 9:
   a. Lower P-CI = $[14.4 – (-3.996*3.7*48^{0.5})]$ = 16.5 minutes
   b. Upper P-CI = $[14.4 – (-5.478*3.7*48^{0.5})]$ = 17.3 minutes

NOTE: the spreadsheet automatically converts the inputted values of the non-central t-percentiles according to the actual λ (that is not displayed); therefore the user must make no manual conversion.

NOTE: the spreadsheet uses the sample size, average and standard deviation information displayed under the "SAMPLE STATISTICS" panel it automatically calculates after the sample data were pasted in.

Therefore, the data manager can found the sought result in the corresponding "RESULT" fields under "PARAMETRIC (GAUSSIAN) CI" panel, or he can directly get it in the narrative result line below where it reads:

"The estimated 75th percentile of the sample is 16.9 minutes (95% P-CI: 16.5 - 17.3)"

Alternatively, the data manager can find out the NP-CI that is computed whereby the same spreadsheet without any further input except the ordered data according to Eq. 13 and Eq. 14 as follows:

a. Lower NP-CI = $(48*0.75) - 1.96*((48*0.75)*(1 - 0.75))^{0.5} = 30$
b. Upper NP-CI = $(48*0.75) + 1.96*((48*0.75)*(1 - 0.75))^{0.5} = 42$

NOTE: it must be recalled that the equations above return the size of the alternative sample partitioning corresponding to the sample quantile, and thus the results shown there must be considered the indexes of the alternative quantile in the original sample.

NOTE: when the dataset contains tied values it should formally more appropriate for indexing data to use ranking instead of simple progressive numbering because it better reflects the discontinue nature of the cumulative binomial function; however, using simple ordering does not change the final result because the NP-CI method relies upon the size of the alternative partitioning and thus on the count of elements expected to fall within it.

Therefore, the data manager can find out the result in the "RESULT" fields under the "NON-PARAMETRIC CI" panel, or he can get it in the narrative result line below where it reads:

"The estimated 75th percentile of the sample is 16.9 minutes (95% P-CI: 15.7 - 18.5)"

For the sake of completeness, the Bias Corrected-accelerated (BCa) bootstrap method with 1,000 re-samples (carried out using an external statistical package) returned the estimate for the 95%CI: 15.9 to 18.4 minutes. Results provided by the three methods are shown in the graph below where the diamond represents the sample estimate of the 75th percentile and the box its 95%CI:

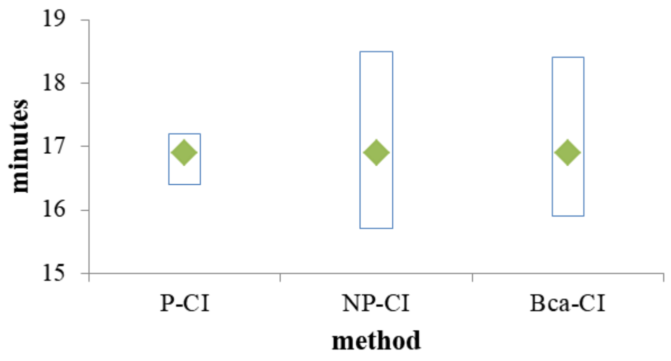

It is evident how the P-CI largely outperforms other non-parametric methods in this case of quasi-Gaussian data returning narrower confidence interval, as well as there is trivial difference between the computationally simple NP-CI and the computationally intensive BCa-CI.
